# Hydroxychloroquine: A Familiar Agent to Combat the Pandemic of COVID-19

**DOI:** 10.3389/fmed.2020.00192

**Published:** 2020-04-30

**Authors:** Vasilios M. Polymeropoulos

**Affiliations:** Vanda Pharmaceuticals, Washington, DC, United States

**Keywords:** COVID-19, hydroxychloroquine, chloroquine, SARS-CoV-2, SARS

## Introduction

The novel coronavirus, COVID-19, has taken the world by storm, causing widespread quarantines and lockdowns of entire nations, actions not seen in over a century since the 1918 Spanish Influenza. The natural defense against an infectious pathogen is to avoid contracting the pathogen altogether through quarantine, a practice employed since antiquity (1). As world leaders allude to the promise of vaccines in a brief time period and the development of novel agents for treatment, perhaps we should examine whether already existing therapeutics are effective in treating people affected with COVID-19.

## Supportive Evidence for Hydroxychloroquine

Researchers in China, the nation first affected by COVID-19, expeditiously assessed the potential use of already existing compounds in treating COVID-19. Hydroxychloroquine and chloroquine (non-hydroxylated compound) have demonstrated effective control of infection with COVID-19 *in vitro* (2). Hydroxychloroquine demonstrated comparable efficacy to chloroquine when used as a treatment against SARS-CoV-2 infection *in vitro* and in Vero cells and was superior to chloroquine when used as pre-treatment prophylaxis (3). Impressive *in vitro* results conducted and published in rapid timeframes have sparked interest into the use of these compounds in the fight against COVID-19 infection, igniting a wave of international clinical trials and leading to the adoption of hydroxychloroquine and chloroquine into national guidelines in several countries, including China, Korea, and Poland, for the treatment of COVID-19 (4).

The potential use of chloroquine or hydroxychloroquine as an antiviral is not a novel concept. Chloroquine has demonstrated efficacy against SARS-CoV *in vitro*, against avian influenza A H5N1 in mice, and against human coronavirus OC43 in newborn mice (5–7), demonstrated long before the emergence of the novel SARS-CoV-2. Although hydroxychloroquine and chloroquine have demonstrated therapeutic antiviral potential in these models, the efficacy of these compounds as antivirals in large controlled clinical studies in humans has yet to be demonstrated.

Clinical evidence is now mounting, as evidenced in a recent clinical study in February 2020 where Gao and colleagues published an interim report from clinical trials describing that chloroquine has demonstrated superior efficacy in the treatment of COVID-19 infection, as seen in multiple disease parameters in humans, leading to a recommendation of its widespread use (8). This report noted significant improvements of chloroquine improving disease parameters including time to seronegative conversion and shortening of disease, though the magnitude of these effects has yet to be published. A clinical trial recently published from Marseille found hydroxychloroquine to be superior to standard of care in eradication of the virus (9). This study had limitations including a small size, a lack of reporting of clinical assessments, and open-label enrollment (9). The results remain promising and are consistent with previous research. These studies are complemented by recent additional reports of encouraging results with hydroxychloroquine or chloroquine in news outlets.

## Discussion

Given that hydroxychloroquine and chloroquine are among the most widely prescribed medications in the world, we can be generally confident about their safety. Of course, with any therapeutic agent there are always risks of adverse effects, and thus the risks and benefits of treatment as always need to be determined on an individual basis. Risks of treatment with hydroxychloroquine include retinal damage (associated with long term use) and QT prolongation. Hydroxychloroquine is contraindicated in people with a history of hypersensitivity to 4-aminoquinoline compounds and should be used with caution in people with glucose-6-phosphate dehydrogenase deficiency (10). Recommended dosing regimens from recent literature, national guidelines, and individual hospital guidelines for COVID-19 have been similar to those currently used for malaria (3, 8). Given that hydroxychloroquine has demonstrated superior efficacy *in vitro* for the prevention of infection, it should be considered as a first-line agent against COVID-19 infection over chloroquine. Hydroxychloroquine should be utilized given the low risks associated with treatment and be further explored as a therapeutic agent at a dose of 400 mg orally per day. In addition to larger clinical studies to evaluate therapeutic efficacy in the setting of active infection, of particular interest would be further studies examining the utility of hydroxychloroquine for prophylaxis against COVID-19 at a weekly dose of 400 mg orally given the long half-life (over 40 days), its previous utility as a prophylactic agent against malaria infections, and promising *in vitro* results (3, 11). Despite limited clinical data, Hydroxychloroquine taken at 400 mg orally per day during active infection may offer an avenue of infection control and treatment of affected individuals in a time of rapid need for therapeutic options. Perhaps the answer to this once in a lifetime pandemic can be found from a dependable agent that has been used in the treatment of malaria for several decades and can be found readily throughout the world.

## Author Contributions

VP completed all work for this manuscript.

## Conflict of Interest

VP was employed by Vanda Pharmaceuticals.

## References

[1] Mackowiak PA Sehdev PS The origin of quarantine. Clin Infect Dis. (2002) 9:1071–2. 10.1086/344062 12398064

[2] Wang M Cao R Zhang L Yang X Liu J Xu M Remdesivir and chloroquine effectively inhibit the recently emerged novel coronavirus (2019-nCoV) *in vitro*. Cell Res. (2020) 3:269–71. 10.1038/s41422-020-0282-0 PMC7054408 32020029

[3] Yao X Ye F Zhang M Cui C Huang B Niu P. *In vitro* antiviral activity and projection of optimized dosing design of hydroxychloroquine for the treatment of severe acute respiratory syndrome coronavirus 2 (sars-cov-2). Clin Infect Dis. (2020). 10.1093/cid/ciaa237. [Epub ahead of print]. 32150618 PMC7108130

[4] Expert consensus on chloroquine phosphate for the treatment of novel coronavirus pneumonia Zhonghua Jie He He Hu Xi Za Zhi. (2020) 3:185–8. 10.3760/cma.j.issn.1001-0939.2020.03.009 32164085

[5] Yan Y Zou Z Sun Y Li X Xu KF Wei Y Anti-malaria drug chloroquine is highly effective in treating avian influenza A H5N1 virus infection in an animal model. Virol J. (2013) 2:300–2. 10.1038/cr.2012.165 PMC3567830 23208422

[6] Vincent MJ Bergeron E Benjannet S Erickson BR Rollin PE Ksiazek TG. Chloroquine is a potent inhibitor of sars coronavirus infection and spread. Virol J. (2005) 2:69. 10.1186/1743-422X-2-69 16115318 PMC1232869

[7] Keyaerts E Li S Vijgen L Rysman E Verbeeck J Van Ranst M Antiviral activity of chloroquine against human coronavirus OC43 inaction in newborn mice. Antimicrob Agents Chemother. (2009) 8:3416–21. 10.1128/AAC.01509-08 PMC2715625 19506054

[8] Gao J Tian Z Yang X Breakthrough: chloroquine phosphate has shown apparent efficacy treatment of COVID-19 associated pneumonia in clinical studies. Biosci Trends. (2020) 1:72–3. 10.5582/bst.2020.01047 32074550

[9] Gautret P Lagier JC Parola P Van Thuan H Meddeb L Mailhe M. Hydroxychloroquine and azithromycin as a treatment of COVID-19: results of an open-label non-randomized clinical trial. Int J Antimicrob Agents. (2020). 10.1016/j.ijantimicag.2020.105949. [Epub ahead of print]. 32205204 PMC7102549

[10] Concordia Pharmaceuticals Inc Plaquenil [package insert]. Saint Michael, MN; Barbados: Concordia Pharmaceuticals Inc (2017).

[11] Tett SE Cutler DJ Day RO Brown KF Bioavailability of hydroxychloroquine tablets in healthy volunteers. Br J Clin Pharmacol. (1989) 6:771–9. 10.1111/j.1365-2125.1989.tb03439.x PMC1379804 2757893

